# Toxicokinetic model of the pyrethroid pesticide lambda-cyhalothrin, main exposure route and dose reconstruction predictions in agricultural workers

**DOI:** 10.1371/journal.pone.0309803

**Published:** 2024-10-23

**Authors:** Jonathan Côté, Michèle Bouchard

**Affiliations:** Department of Environmental and Occupational Health, Chair in Toxicological Risk Assessment and Management, and Public Health Research Center (CReSP), University of Montreal, Montreal, Quebec, Canada; CIFRI: Central Inland Fisheries Research Institute, INDIA

## Abstract

A toxicokinetic model of the pyrethroid insecticide lambda-cyhalothrin (LCT) was developed to relate absorbed doses to urinary cis-3-(2-chloro-3,3,3-trifluoroprop-1-en-1-yl)-2,2-dimethylcyclopropanecarboxylic acid (CFMP) metabolite levels used as a biomarker of exposure. The model then served to reconstruct absorbed doses in agricultural workers and their probability of exceeding the EFSA *Acceptable occupational Exposure Level* (AOEL). The toxicokinetic model was able to reproduce the temporal profiles of CFMP in the urine of operators spraying pesticides using the optimized model parameters (adjusted to human volunteer data). Modeling also showed that simulation of an inadvertent oral exposure mainly was the exposure scenario giving the best fit to CFMP urinary time-course data in applicators. With the dermal model parameters optimized from data in volunteers, simulation of a dermal exposure in applicators did not allow to reproduce the observed peak excretions and urinary metabolite levels; extremely high applied dermal doses would be required but still simulated dermal penetration rate would remain too slow. Simulation of an inhalation exposure allowed to reproduce the observed time-courses, but with unrealistic air concentrations. For applicators with the highest urinary concentrations, there was a probability of exceeding the AOEL at some points during the biomonitoring period [>50% probability of exceeding for 27% of 24-h samples]; for non-applicator workers the probability of exceeding the AOEL value was very low [corresponding value of 5%]. Furthermore, the median [95% CI] estimates of 10 000 Monte Carlo simulations led to a biological reference value corresponding to the AOEL of 116 [113–119] ng/kg bw/d and 7.5 [7.3–7.7] μg/L. Overall, 7% of applicators and 1% of workers performing weeding and strawberry picking had a probability of exceeding this biological reference value. As a next step, it would be interesting to apply these methods to multiple exposure to various contaminants.

## Introduction

Pyrethroid pesticides are among the most widely used insecticides in agricultural settings to control insect pests [1–4]. They are applied extensively to various crops (vegetables, fruits, corn) such that agricultural workers are exposed on a regular basis. Lambda-cyhalothrin (LCT) is among the pyrethroids extensively used on these crops. This fast-acting contact and ingestion insecticide is effective on a wide range of pests. It is also widely used to control pests and parasites in buildings and their perimeters [5].

LCT is known to have an impact on the endocrine and immune systems of rats, as well as causing neurological and motor effects [6–8]. In humans, neurological effects were documented in cases of LCT poisoning [9]. LCT may also have effects on human thyroid and reproductive hormone levels [10]. In order to adequately characterize pesticide exposure in workers, it is necessary to develop methods that determine actual absorbed doses. Biological monitoring of exposure is thus conducted by measuring pesticide metabolites in urine [1, 11]. Nevertheless, interpretation of biological monitoring data requires a good understanding of the fate (toxicokinetic behavior) of the substance of interest in the human body in order to establish appropriate links between levels of biomarkers of exposure in workers and doses actually absorbed.

To help make this link, biomathematical models can be developed to simulate the fate of the compounds of interest and their biomarkers in the body. In these models, the body is represented as compartments; each compartment represents a tissue or a set of tissues. The rate of change in the amounts of the chemical compound and its metabolites in each compartment is then determined by the difference between the amounts entering and leaving the compartment per unit of time. Only the key processes describing the kinetics in the selected biological matrices are determined. The transfer coefficients between the different compartments thus describe global physiological phenomena. The overall processes involved in the intercompartmental transfer rate include phenomena such as, but not limited to, first-pass metabolism, differences between metabolizing enzymes and other metabolism-related phenomena, retention in different tissues and renal elimination. These models can simulate different exposure scenarios and different routes of uptake by workers. They can thus be used to reconstruct the absorbed doses of a compound from biomarker measurements in matrices such as urine, for different exposure scenarios.

Some research groups have developed physiologically-based pharmacokinetic (PBPK) models for certain pyrethroid pesticides [12–18]. Simple compartment models were also used to relate urinary levels to doses and infer on urinary levels corresponding to a critical dose in the European consortium "Human Biomonitoring for Europe" (HBM4EU) [19]. The work carried out in recent years has further led to the development of toxicokinetic modelling tools to better interpret biomonitoring of exposure data to priority pesticides in agricultural workers [20–23]. These models were built using kinetic data in human volunteers exposed under controlled conditions where the doses are known and the critical biological determinants of the observed biological levels (including intra- and inter-individual variability) can be well identified. Such toxicokinetic model has been used for dose reconstruction of the pyrethroids cypermethrin and permethrin based on urinary measurements of metabolites [20]; these kinetic data in volunteers served for model conceptual and functional representation as well as the determination of model parameters [24, 25]. Only human kinetic data were used with this modeling approach considering the known differences in the kinetics of pyrethroids between animals and humans [24, 25]. This modeling approach can be adapted to simulate the kinetics of other pyrethroids, such as LCT, and to relate absorbed doses and urinary metabolite levels used as biomarkers of exposure.

**Fig 1:**
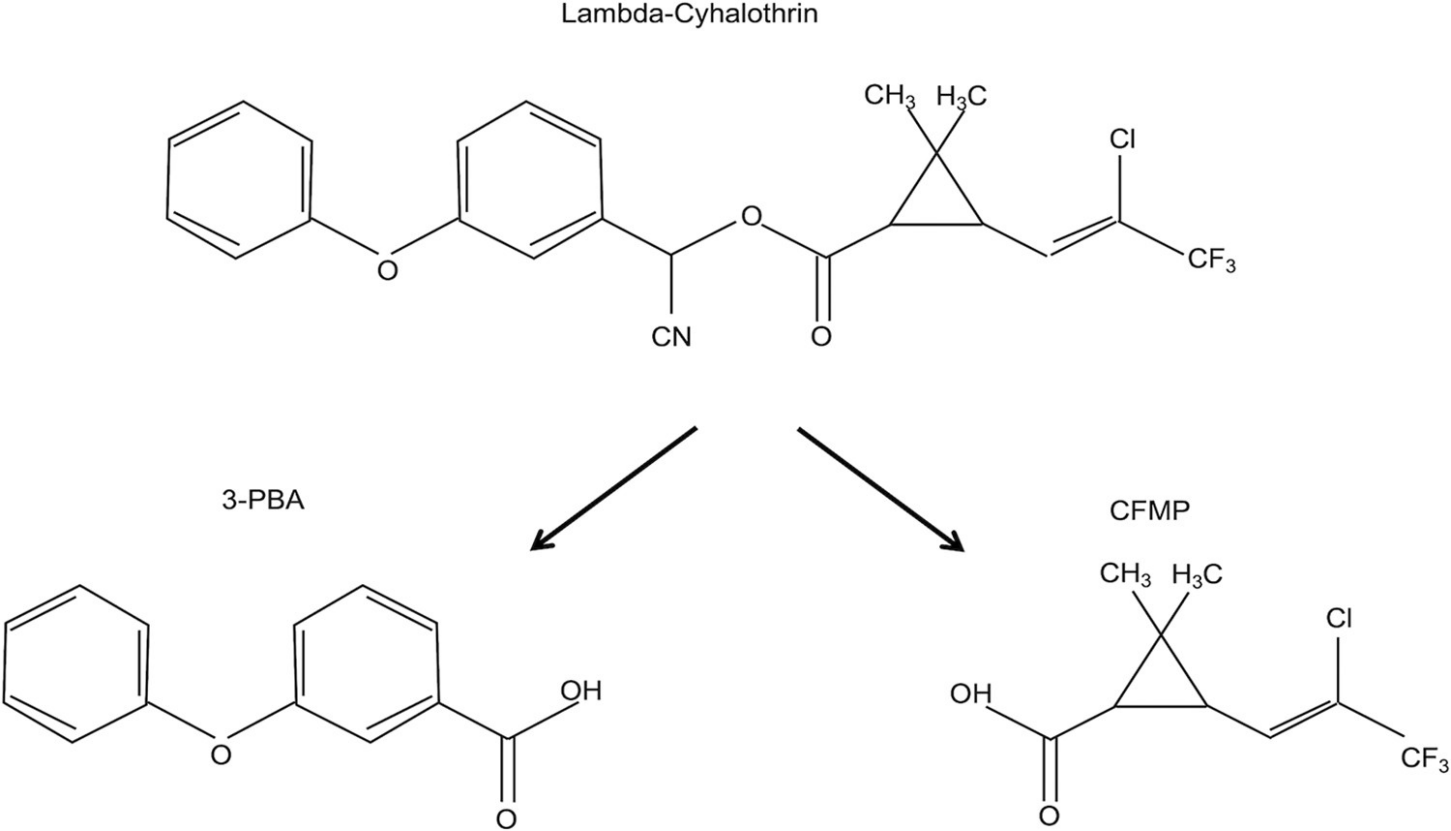
Representation of the metabolism of LCT into main metabolites used as biomarkers of exposure.

LCT has been shown to be rapidly split in the body, by carboxylesterases and P450 cytochromes, to generate several metabolites [26–29], but it is metabolized into two major metabolites used as biomarkers of exposure (see Fig 1). These metabolites are excreted in urine and feces in the days following exposure [26, 30]. One of the main urinary metabolites of LCT is 3-phenoxybenzoic acid (3-PBA) (CAS number 3739-38-6), also common to other pyrethroids (including permethrin, cypermethrin and deltamethrin); it is readily detected in the urine and measured in biomonitoring studies in workers and individuals of the general population [31–37]. Another major metabolite of LCT, measured more recently in biomonitoring studies, is cis-3-(2-chloro-3,3,3-trifluoroprop-1-en-1-yl)-2,2-dimethylcyclopropane carboxylic acid (CFMP; otherwise known as ClF_3_CA) (CAS number: 72748-35-7) [38, 39]. This CFMP metabolite is now being quantified in the large population-based biomonitoring survey of exposure to various contaminants of the HBM4EU [19, 40–44]. 3-PBA and CFMP measurements in urine can thus be used to reconstruct absorbed doses in exposed individuals using toxicokinetic modelling.

Such toxicokinetic model can also serve to establish urinary levels corresponding to a critical toxicological reference dose. In the context of preventing risks associated with occupational exposure, the European Food Safety Authority (EFSA) [45] has published a systemic *Acceptable Operator Exposure Level* (AOEL) of 0.00063 mg/kg bw/day. This EFSA toxicological reference value is based on a no-observed adverse effect level (NOAEL) of 0.5 mg/kg bw/day in a multigeneration study in rats exposed to cyhalothrin, to which an uncertainty factor of 200 has been applied (the standard factor of 100 for a chronic animal study and an additional factor of 2 for the conversion from cyhalothrin to LCT) and considering a limited absorption fraction of 25% [45]. This AOEL corresponds to a maximum internal (absorbed) dose limit that should not be exceeded to prevent long-term adverse effects in repeatedly exposed operator workers involved in activities related to pesticide application (mixing/loading of product into machinery, operation of machinery, including repair and cleaning after use) [46]. The objective of the current study was thus to develop a toxicokinetic model to reconstruct absorbed doses of LCT based on metabolite measurements used as biomarkers of exposure. A second objective was to derive a biological reference value corresponding to the available AOEL established by EFSA.

## Materials and methods

### Ethics and published peer-reviewed data used for modeling

The study protocol including the use of secondary published data for the modeling, as well as other relevant documents were approved by the Research Ethics Committee of the Université de Montréal (approval CERC-19-0007-D, on 06-03-2019). Published data from our research group were used for modeling in the current work; subjects who participated in the published studies (all adults) signed a free and informed written consent form after receiving all necessary information about the project. Each participant was free to withdraw at any time. The anonymity of the subjects was also respected by coding the samples.

The published data of Khemiri et al. [27, 28]–from our laboratory–on the blood and urinary profiles of the metabolites CFMP and 3-PBA observed in volunteers exposed orally and dermally to LCT were used to build the toxicokinetic model and determine its parameters (see S1 Appendix for methodological details).

The biomonitoring data from the field study of Bossou et al. [39] on the kinetics of CFMP served to reconstruct absorbed doses of LCT in strawberry farmworkers using the toxicokinetic model developed in the current work (see S1 Appendix for details).

### Development of the toxicokinetic model and dose reconstruction

#### Toxicokinetic modeling for the simulation of the kinetic profiles of LCT in volunteers

**Fig 2:**
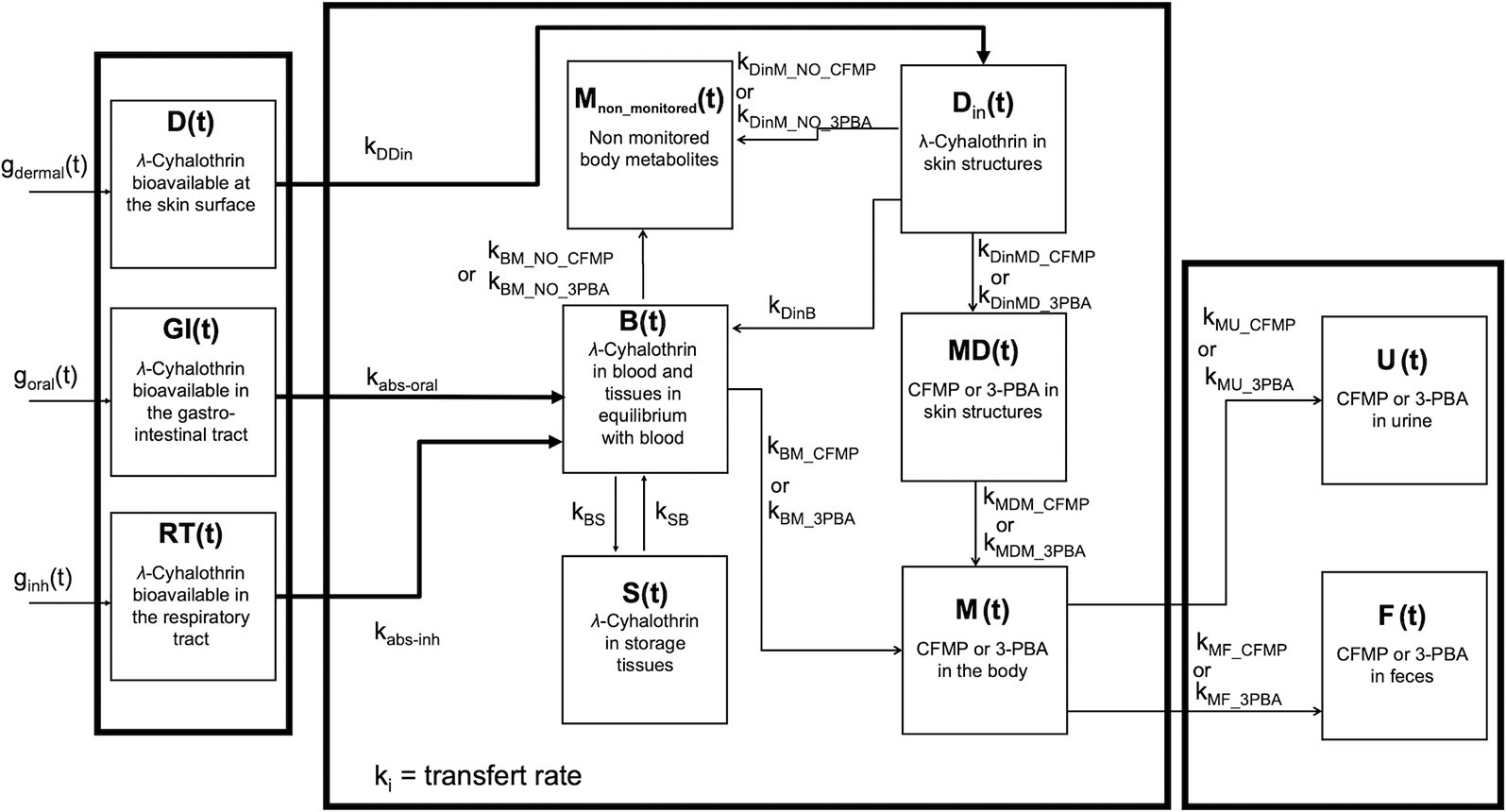
Conceptual model of the kinetics of LCT and its metabolites used as biomarkers of exposure.

A toxicokinetic model specific to LCT was developed to simulate the kinetic time course of metabolites used as biomarkers of exposure in individuals exposed to LCT by oral, inhalation and dermal routes. This type of model translates, in mathematical terms, the essential determinants of the temporal evolution of the pesticide and its metabolites in the human body (Fig 2 and Table 1). The model can be used to reproduce the temporal profiles of exposure biomarkers in urine and infer on the corresponding absorbed doses. In the developed model, the distribution of LCT and its biotransformation products were represented by transfers from one compartment to another at rates proportional to the source compartment burden. The rate of change in amounts in each compartment (dXi(t)/dt) (on a molar basis) is therefore the difference between the amounts entering and leaving the compartment per unit time. Specific compartments, D(t), GI(t), RT(t), were used to describe the amounts of LCT available at the skin surface, in the lumen of the gastrointestinal tract, and in the respiratory tract, respectively. The blood burden and the tissue burden of LCT rapidly in equilibrium with blood burden were grouped into a single compartment B(t) since these amounts change in parallel. A LCT storage compartment S(t) was also introduced into the model to describe accumulation in lipids or binding to tissue proteins. This compartment S(t) was inserted into the model to account for the biphasic elimination of LCT metabolites in orally exposed volunteers [27]. A M(t) compartment was used to represent body burdens of the CFMP or 3-PBA metabolites as a function of time. A compartment M_non_monitored_(t) was inserted to represent the body burden of unmeasured metabolites in blood and urine. A U(t) compartment was used to represent the cumulative excretion of CFMP or 3-PBA in urine and F(t) compartment served to represent the cumulative fecal excretion of CFMP or 3-PBA as a function of time. To account for differences in the kinetics of the CFMP and 3-PBA metabolites in the matrices of dermally exposed volunteers, a D_in_(t) compartment was added to account for the burden of LCT absorbed into the skin structures. A MD(t) compartment was also added to describe LCT amounts metabolized to CFMP and 3-PBA within the skin structures.

**Table 1:** Description of the symbols used for the conceptual and functional representation of the kinetic model of LCT and its metabolites.

Parameter	Definition
g_oral_ (t) g_dermal_(t) g_inh_ (t)	Oral dose (mol) per unit time that can describe temporal variations in inputs Skin dose (mol) per unit time that can describe temporal variations in inputs Inhalation dose (mol) per unit time that can describe temporal variations in inputs
D(t) D_in_ (t)	Amounts of LCT (mol) available at the skin surface as a function of time Amounts of LCT (mol) inside the skin structures as a function of time
GI(t)	Amounts of LCT (mol) available in the gastrointestinal tract as a function of time
RT(t)	Amounts of LCT (mol) available in the respiratory tract as a function of time
B(t)	LCT burden (mol) in blood and in tissues in dynamic equilibrium with blood as a function of time
S(t)	LCT burden (mol) retained in tissues (mol) as a function of time
M(t) MD(t) M_non_monitored_ (t)	Body burden of CFMP or 3-PBA (mol) as a function of time Burden of CFMP or 3-PBA (mol) inside the skin structures as a function of time Body burden of non monitored metabolites (mol) as a function of time
U(t)	Cumulative amounts of CFMP or 3-PBA in urine (mol) as a function of time
QU(t)	Urinary excretion rate of CFMP or 3-PBA in urine (mol) as a function of time = M(t) x k_MU_
F(t)	Cumulative amounts of CFMP or 3-PBA in feces (mol) as a function of time
f_abs_oral_	Oral absorption fraction of LCT
f_abs _dermal_	Dermal absorption fraction of LCT
k_abs_oral_	Oral absorption rate of LCT (h^-1^)
k_DDin_	Dermal absorption rate of LCT to internal skin structures (h^-1^)
k_abs_inh_	Respiratory absorption rate of LCT (h^-1^)
k_DinB_	Transfer rate of LCT from internal skin structures to blood (h^-1^)
k_BS_	Transfer rate of LCT from blood to storage tissue (h^-1^)
k_SB_	Transfer rate of LCT from storage tissue to blood (h^-1^)
k_BM_CFMP_ k_BM_3PBA_	Hybrid biotransformation rate of LCT to CFMP (h^-1^) Hybrid biotransformation rate of LCT to 3-PBA (h^-1^)
k_DinMD_CFMP_ k_DinMD_3PBA_	Hybrid biotransformation rate of LCT to CFMP in internal skin structures (h^-1^) Hybrid biotransformation rate of LCT to 3-PBA in internal skin structures (h^-1^)
k_MDM_CFMP_ k_MDM_3PBA_	CFMP transfer rate from internal skin structures to the body (h^-1^) Transfer rate of 3-PBA from internal skin structures to the body (h^-1^)
k_BM_NO_CFMP_ and k_DinM_NO_CFMP_ k_BM_NO_3PBA_ and k_DinM_NO_3PBA_	Biotransformation rate of LCT to non monitored metabolites derived from CFMP (h^-1^) Biotransformation rate of LCT to non monitored metabolites derived from the phenoxybenzoic form (h^-1^)
k_MU_CFMP_ k_MU_3PBA_	Transfer rate of CFMP from the body to urine (h^-1^) Transfer rate of 3-PBA from the body to urine (h^-1^)
k_MF_CFMP_ k_MF_3PBA_	Transfer rate of CFMP from the body to feces (h^-1^) Transfer rate of 3-PBA from the body to feces (h^-1^)

The model also assumes the absence of saturation in the metabolism and clearance processes. The kinetic profiles of CFMP and 3-PBA were accurately predicted in volunteers without introducing saturation [27, 28]. In animals exposed to a very high dose of LCT intravenously (3 mg/kg bw) or orally (20 mg/kg bw), no saturation was apparent based on the time profile of the parent compound [26]. However, the model cannot be used to predict kinetics at saturating exposure doses.

#### Determination of model parameters

The differential equations of the functional representation of the toxicokinetic model were solved using a matrix resolution (see S2 Appendix), which allowed to obtain mathematical descriptions of the kinetic time course of the compounds of interest (amounts of CFMP and 3-PBA as a function of time in our case) for each compartment of the model. The programming also allowed simulation of single, multiple or serial exposures for any type of oral, dermal or inhalation exposure. The matrix resolution was combined with a built-in function for the optimization of model parameters in order to allow a fit to the observed temporal profile data. This was done using the *nonlinear* least square *solver* (LSQNonLin) in Matlab (Matlab version R2022a, MathWorks, Natick, Massachusetts, USA). This combined computer programming offers both robustness in the determination of the kinetic parameters and a substantial improvement in the resolution speed over conventional numerical methods. The model parametric value search relies on determining 1000 sets of acceptable parametric values for each available set of observed time course data. The computer routine randomly selects initial values (within a given range) and then varies the set of parametric values in an attempt to fit the simulated profiles to the observed data by the least squares method. These iterations are repeated until 1000 sets of parametric values are obtained, with a maximum error of 20% on fits to the observed profiles. Thus, for the simulation of a given set of data, each parametric value is defined with a standard deviation. (See S1 Fig for the algorithm used for the determination of the model parametric values).

A distribution of parametric values for each model parameter was first determined from the data of Khemiri et al. [27] on the individual time courses of CFMP in blood and urine of the seven orally exposed volunteers along with the average values for all volunteers as a function of time. All the parameters representing the kinetics of absorbed LCT and its CFMP and 3-PBA metabolites (see Table 1) were determined from fits to these oral sets of data. Parameters related to dermal absorption were then estimated using the data of Khemiri et al. [28] on the time courses of CFMP in blood and urine of four of the six volunteers exposed dermally to Matador^®^. For the other two volunteers, the values were too low to allow modeling. To establish the dermal-related parameters (f_abs _dermal_, k_DDin_, k_DinB_, k_DinMD_CFMP_, k_DinMD_3-PBA,_ k_MDM_CFMP,_ k_MDM_3-PBA_), the sets of parameter values determined from fits to the individual time course of metabolites in the orally exposed volunteers were used in the model; only the dermal-specific parameters were then determined. Again, the sets of dermal-specific parametric values retained were those that gave a good fit to each of the individual kinetic profiles of the dermally exposed volunteers (a profile for each volunteer). For the inhalation scenario, the transfer constant from the lungs to the blood was considered very fast and was set by default at a half-life of 1 minute.

The complete resolution of the system of differential equations in Matlab with the optimized sets of parametric values allowed the simulation of the time courses of LCT and its metabolites CFMP and 3-PBA in the different compartments. This includes simulations of the urinary excretion time courses of metabolites considering various exposure scenarios.

#### Dose reconstruction in strawberry field pesticide applicators and workers

Once the model was developed and its parametric values determined, it was used to reconstruct absorbed doses from urinary excretion time courses of metabolites observed in workers. This was done by taking into consideration different exposure routes and times. Absorbed dose reconstruction was performed in Matlab (Matlab version R2022a, MathWorks, Natick, Massachusetts, USA). The simulated exposure scenarios were based on answers to the self-administered questionnaires during each of the biomonitoring days. An exclusively dermal exposure scenario was first evaluated. In this case, a calculation was made to determine the amounts expected to be found on the skin through direct contact with pure or diluted Matador^®^ or through indirect contact with strawberry leaves following application. Matador 120EC^®^ contains 120 g/L LCT, *i*.*e*. 0.27 mol/L. The application rate is 104 mL/ha (1.04 nL/cm^2^) or 12.48 g/ha [5]. To estimate the number of leaves handled, a hypothetical surface area of 100 cm^2^ per strawberry leaf was used; this would be equivalent to 0.104 μL of Matador^®^ per leaf or 28 nmol of LCT. According to Brouwer et al. [47], when the whole hand comes into contact with a contaminated surface, the transfer of contaminant from the surface to the hand is 2%.

An inhalation exposure scenario was also considered. Reconstructed doses were determined on an hourly average basis. In the case of inhaled dose reconstruction, a calculation was also made to estimate the airborne concentration of LCT, considering an overestimated absorption fraction of 100%, a respiratory frequency of 20 breaths per minute and a volume of 500 mL per breath [48].

An oral exposure scenario by ingestion of LCT residues from contaminated food was further simulated (considering food consumption times reported by questionnaire). In addition, oral exposure scenarios mimicking hand-to-mouth inadvertent ingestion were simulated. In the case of applicators and potential exposure through inadvertent ingestion, hand-to-mouth behavior, an hourly average exposure was considered. This scenario can also include the consumption of contaminated food or any form of oral contamination, as it reconstructs hourly absorbed doses over the entire follow-up. A similar principle was applied to simulate the excretion time course of metabolites in the other field workers performing weeding and strawberry picking tasks and for which consecutive 24-h urine collections were performed; however, five oral exposures were simulated per day to mimic hand-to-mouth inadvertent ingestion or any other form of oral contamination. For field workers, preliminary tests showed that modeling beyond five daily oral doses had no significant impact on the simulated 24-h urinary excretions of CFMP, in contrast with simulations of continuous exposure in the applicators for which serial samples were collected.

Reconstruction of the absorbed doses for each worker was performed by adjustments to measured amounts of CFMP metabolites in workers for each (time) period of urine collection, over the whole biomonitoring period. The reconstruction of absorbed doses was based on urinary measurements of the CFMP metabolite, given that it is more specific to LCT. Considering the reconstructed absorbed doses for each worker, the probability of exceeding the available AOEL reference dose of 0.00063 mg/kg bw/d (1400 pmol/kg bw/d) derived by EFSA [45] was calculated. Specifically, a Monte Carlo simulation was programmed, where absorbed doses were reconstructed based on a lognormal distribution of the previously determined sets of parameter values. Thus, for each worker, a Monte Carlo simulation was performed to obtain the reconstructed absorbed dose possibilities for each simulated exposure time. A limit was imposed on the value of the least-squares minimization result R. Any result exceeding a R value corresponding to a maximum average deviation of 5% between the observed points and the corresponding simulations was excluded. The Monte Carlo simulation was run until 1000 reconstructed absorbed dose profiles corresponding to exposure scenarios meeting the inclusion criterion were obtained. The reconstructed daily doses for the applicators (*i*.*e*., the summation of absorbed doses per period of time over a 24-h period) and the other farmworkers performing weeding or strawberry picking were compared to the AOEL reference value. From the 1000 dose values reconstructed for the same worker at each 24-h period, the percentage of values exceeding the AOEL daily reference dose was calculated to represent the probability of exceeding the reference value on a daily basis. It should be noted that the Monte Carlo method described above stops at 1000 results, as preliminary tests have shown that beyond this number, the probability of exceeding the AOEL daily dose remains the same. (See S2 Fig for the algorithm used for dose reconstruction in workers).

In addition, the model was used to derive a biological reference value of CFMP corresponding to urinary amounts in a 24-h collection (ng CFMP/kg bw/d) obtained by simulating repeated exposures to the AOEL of 0.00063 mg/kg bw/d until a steady state was reached, *i*.*e*. an hourly dose of 1/8 of the AOEL over an 8-h period per day repeated over five days. A total of 10 000 Monte Carlo simulations were performed by randomly selecting 10 000 sets of parametric values according to a lognormal distribution. A biological reference concentration of CFMP in μg/L urine was also derived by considering the mean urine volume and mean body weight calculated in the study workers.

## Results

### Optimization of the toxicokinetic model parameters

**Fig 3:**
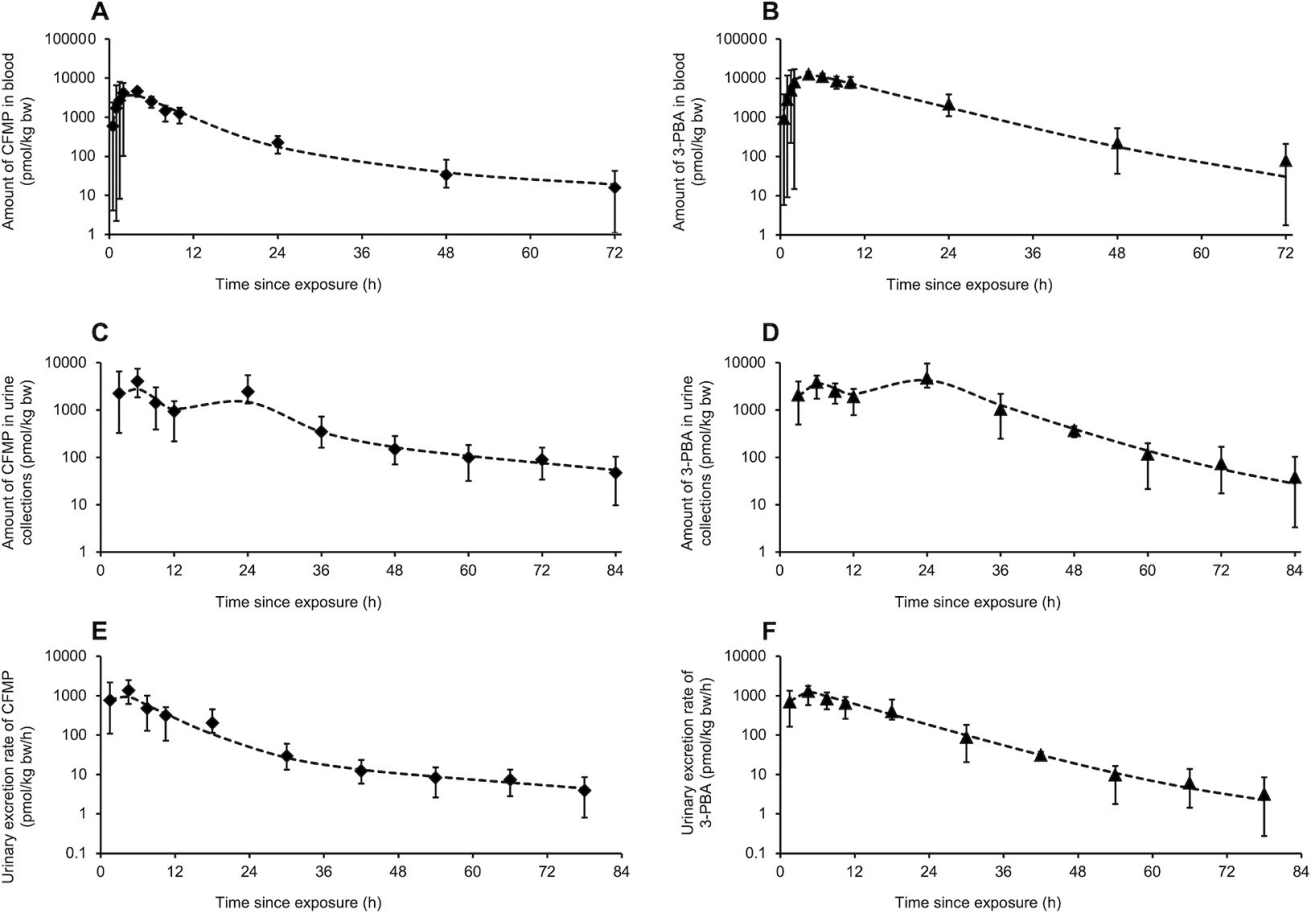
Comparison of model simulations (----) to observed data of Khemiri et al. [27] on the temporal profiles of CFMP (*◆*) and 3-PBA (▯) (mean ± SD) in blood and urine of volunteers orally exposed to LCT. A and B; CFMP and 3-PBA in blood in pmol/kg bw. C and D; CFMP and 3-PBA in urine collections in pmol/kg bw. E and F; Urinary excretion rate of CFMP and 3PBA in pmol/kg bw/h.

**Fig 4:**
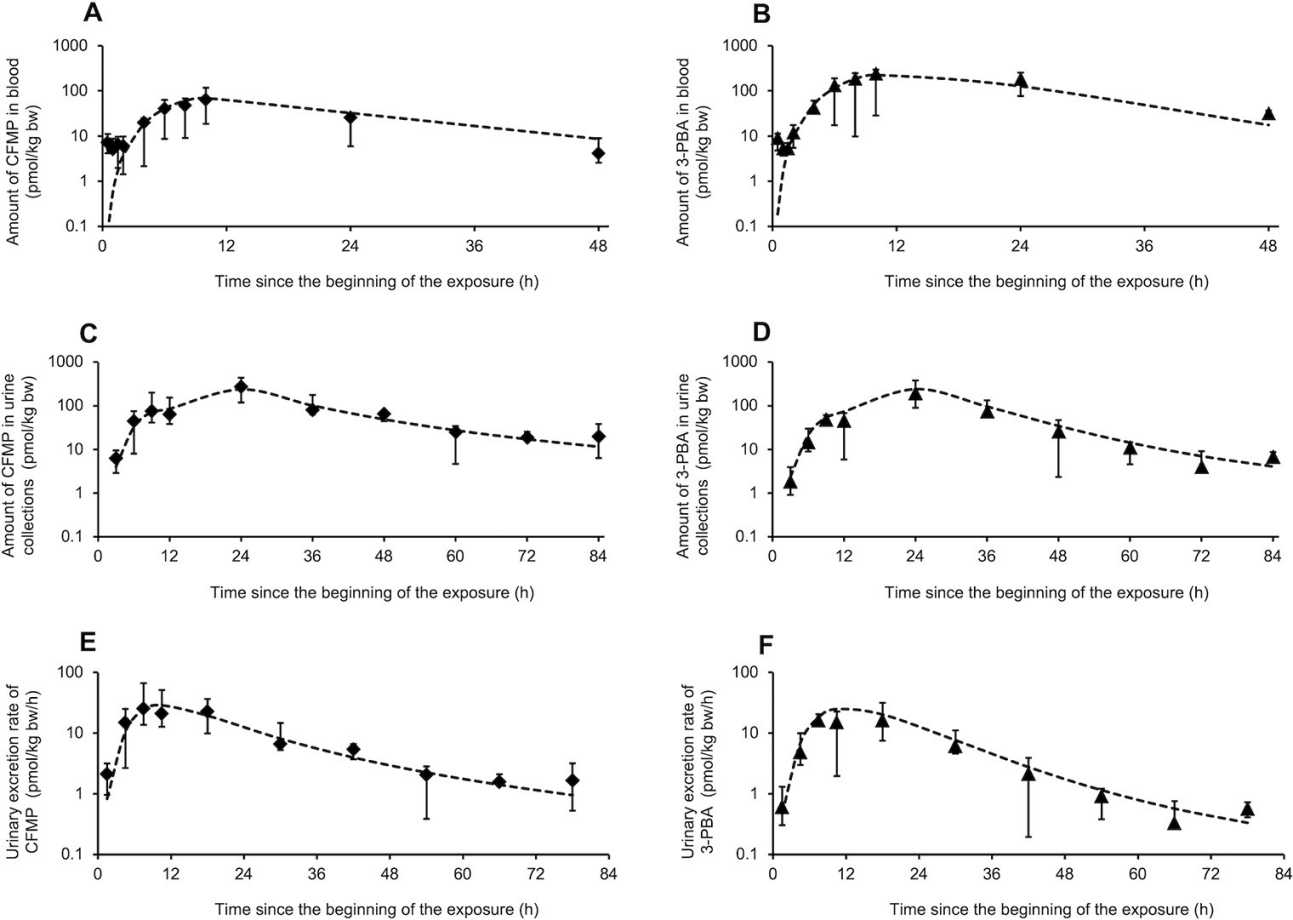
Comparison of model simulations (----) to observed data of Khemiri et al. [28] on the temporal profiles of CFMP (*◆*) and 3-PBA (▯) (mean ± SD) in blood and urine of volunteers dermally exposed to LCT. A and B; CFMP and 3-PBA in blood in pmol/kg bw. C and D; CFMP and 3-PBA in urine collections in pmol/kg bw. E and F; Urinary excretion rate of CFMP and 3PBA in pmol/kg bw/h.

Tables 2 and 3 show the toxicokinetic model parameters derived from adjustments to the observed data of the Khemiri et al. [27, 28] on the temporal profiles of CFMP and 3-PBA in blood and urine of volunteers exposed orally and dermally to LCT under controlled conditions. With these optimized parameters, the model gave an adequate approximation to the observed time courses in the volunteers as illustrated in Figs 3 and 4.

**Table 2:** Toxicokinetic model parameters optimized by adjustments to the observed kinetic data of Khemiri et al. [27] in volunteers orally exposed to LCT.

	Oral absorption fraction and transfer constants (h^-1^) (mean ± SD) of the model											
	LCT				CFMP				3PBA			
Volunteers	f_abs_oral_	k_abs_oral_	k_BS_	k_SB_	k_BM_CFMP_	k_BM_NO_CFMP_	k_MU_CFMP_	k_MF_CFMP_	k_BM_3PBA_	k_BM_NO_3PBA_	k_MU_3PBA_	k_MF_3PBA_
1	0.88 ± 0.18	2.3 ± 0.54	0.29 ± 0.09	0.08 ± 0.00	0.50 ± 0.36	1.5 ± 0.15	0.29 ± 0.02	0.03 ± 0.04	1.3 ± 1.2	0.92 ± 0.42	0.12 ± 0.004	0.02 ± 0.01
2	0.96 ± 0.06	2.3 ± 0.05	0.06 ± 6E-4	0.002 ± 1E-5	0.03 ± 0.002	0.03 ± 0.002	0.40 ± 0.002	0.03 ± 0.01	0.09 ± 0.01	0.13 ± 0.01	0.11 ± 0.002	0.01 ± 0.003
3	0.79 ± 0.08	0.89 ± 0.06	0.20 ± 0.06	0.07 ± 0.004	0.13 ± 0.02	0.46 ± 0.16	0.25 ± 0.01	0.13 ± 0.38	0.80 ± 0.15	0.95 ± 0.13	0.08 ± 0.01	0.04 ± 0.01
4	0.92 ± 0.16	1.3 ± 0.56	0.05 ± 0.07	0.02 ± 0.002	0.08 ± 0.10	0.33 ± 0.41	0.26 ± 0.01	0.007 ± 0.01	0.45 ± 0.59	0.31 ± 0.38	0.09 ± 0.003	0.04 ± 0.01
5	0.88 ± 0.16	1.4 ± 0.46	0.08 ± 0.04	0.04 ± 0.003	0.32 ± 0.22	0.48 ± 0.18	0.60 ± 0.03	0.002 ± 6E-5	0.40 ± 0.23	0.43 ± 0.17	0.13 ± 0.003	0.06 ± 0.07
6	0.89 ± 0.10	0.92 ± 0.06	0.15 ± 0.05	0.08 ± 0.02	0.16 ± 0.02	0.46 ± 0.16	0.32 ± 0.01	0.10 ± 0.30	0.46 ± 0.09	0.72 ± 0.11	0.09 ± 0.003	0.02 ± 0.01
7	0.94 ± 0.12	0.90 ± 0.02	0.07 ± 0.02	0.04 ± 0.01	0.13 ± 0.03	0.61 ± 0.05	0.22 ± 0.002	0.002 ± 4E-5	0.36 ± 0.06	0.63 ± 0.23	0.06 ± 0.01	0.04 ± 0.02

**Table 3:** Toxicokinetic model parameters optimized by adjustments to the observed kinetic data of Khemiri et al. [28] in volunteers dermally exposed to LCT.

	Transfer constants (h^-1^) (mean ± SD) of the model							
	LCT		CFMP			3PBA		
Volunteers	k_DDin_	k_DinB_	k_DinM_NO_CFMP_	k_DinMD_CFMP_	k_MDM_CFMP_	k_DinM_NO_3PBA_	k_DinMD_3PBA_	k_MDM_3PBA_
2	0.0005 ± 1E-6	0.49 ± 0.01	0.0001 ± 6E-6	0.03 ± 0.01	0.0530 ± 0.0002	0.0002 ± 0.0007	0.09 ± 7E-6	0.0001 ± 5E-7
3	0.0005 ± 9E-5	0.12 ± 0.01	0.0003 ± 0.0008	0.10 ± 0.03	0.0001 ± 6E-7	0.0001 ± 7E-6	0.03 ± 0.03	0.10 ± 0.10
5	0.0009 ± 0.0002	0.08 ± 0.02	0.0001 ± 3E-5	0.004 ± 0.04	0.08 ± 1.1	0.0001 ± 0.0006	0.13 ± 0.03	0.0002 ± 8E-5
6	0.0004 ± 8E-5	0.03 ± 0.01	0.0001 ± 1E-6	0.09 ± 0.01	0.002 ± 0.005	0.0002 ± 0.001	0.03 ± 0.004	0.0001 ± 0.05

### Simulation of the temporal profiles in applicators, reconstruction of absorbed doses and probabilities of exceeding the AOEL

**Fig 5:**
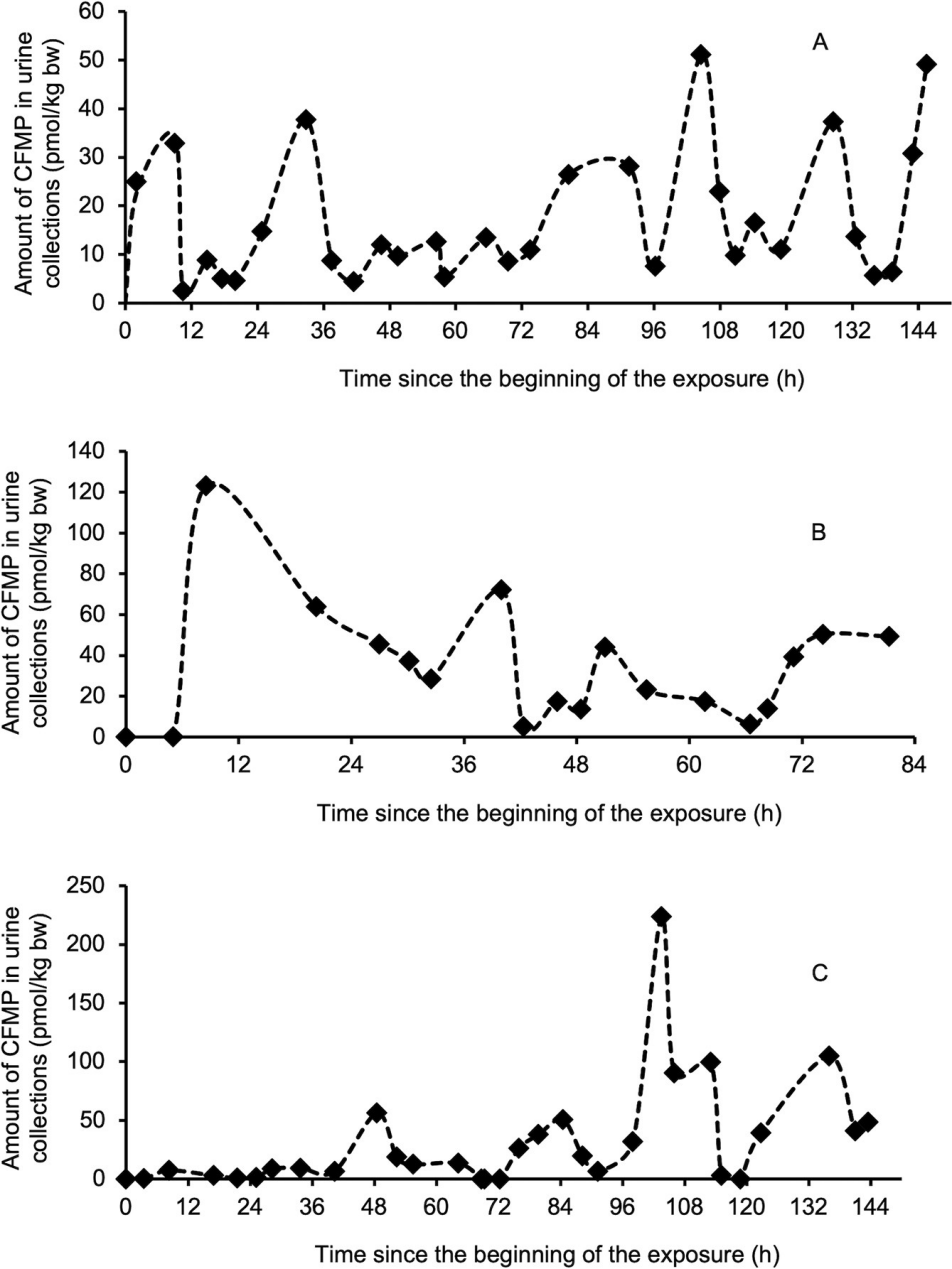
Comparison of model simulations (----) with observed data on the excretion time courses of CFMP (*◆*) in the urine of applicators. A) T101 and exposure scenarios E2 and E3; B) T103 and exposure scenario E1; C) T106 and exposure scenarios E2 and E3. The dots represent model simulations of amounts excreted at each period and the dotted line represents the trend in excretion time courses.

With the model parameters optimized by adjustment to the data of Khemiri et al. [27, 28] in volunteers exposed under controlled conditions, the toxicokinetic model was able to reproduce the data of Bossou et al. [39] on the temporal profiles of the more specific CFMP metabolite of LCT observed in the urine of the operators spraying pesticides. Fig 5 depicts examples that show that the model gives an excellent fit to the observed urinary excretion time courses of CFMP in applicators, after performing the Monte Carlo simulation generating 1000 sets of parametric values and giving a fit to the observed data with a margin of error of less than 5%. The modeling also showed that it was possible to have an excellent fit to the observed urinary excretion time courses of CFMP in applicators both after exposure to LCT alone and after co-exposure with captan, without having to modify the model parameters and thus without considering any interaction effect. The modeling also showed that a simulation of inadvertent hourly oral exposure mainly was the exposure scenario that gave the best fit to the excretion time courses of metabolites observed in urine of the applicators. With the dermal parameters optimized from data in volunteers, the model could not adequately reproduce the observed temporal profiles in applicators based primarily on dermal absorption inputs, as simulated dermal penetration rate was too slow for the observed peak excretion. Also, extremely high applied dermal doses, *i*.*e*. orders of magnitude higher than the AOEL, would be required to give a good fit to observed urinary metabolite levels. To verify the absence of significant dermal absorption, we estimated the dermal exposure that would be necessary to reach peak excretion rates of CFMP in workers. For instance, peak excretion rate of CFMP in worker T106 was 17 pmol/kg bw/h at times 72–91 h; this value corresponds to around 63% of maximum levels observed in volunteers exposed to Matador^®^ and a LCT active ingredient dose of 0.25 mg/kg bw (0.56 μmol/kg bw) via the dermal route in the study of Khemiri et al. [28]. Multiplying this factor by the dose applied in volunteers would give a dermal dose of 0.35 μmol/kg bw or 33.3 μmol of LCT for this T106 worker weighing 95 kg. Worker T106 would therefore unrealistically have to be exposed to around 124 μL of pure Matador, or at least 300 mL of diluted Matador over a 6-h period. Assuming a 100% transfer of LCT from strawberry leaves to the skin, around 1200 entire strawberry leaves would have to be handled. For a 2% transfer, this number would be around 60 000 entire strawberry leaves.

Simulation of an inhalation exposure also allowed to give a good fit to the observed urinary excretion time courses of CFMP in applicators. However, estimated air concentrations were unrealistic, even when considering the lowest estimated air concentrations needed to provide a good fit to excretion profiles based on 1000 sets of plausible reconstructions (*i*.*e*., representing the scenario producing the lowest air concentrations). For instance, worker T106 would have to be exposed to a median hourly concentration of 0.36 μg/m^3^ with maximums of 9.3 μg/m^3^ to allow an adequate simulation of excreted CFMP. These calculations exclude hours with no exposure. For comparison purposes with studies measuring insecticide concentrations in air, these concentrations are equivalent to a median of 29 μg/m^3^/AR 1 kg AI/ha with maximums at 744 μg/m^3^/AR 1 kg AI/ha (where AR represents the application rate and AI the active ingredient [49]). More precisely, the urinary excretion time course of CFMP in worker T106 shows a very slight increase in urinary levels 8 h after application of LCT. Assuming an absorption solely by inhalation, atmospheric concentrations would have to average 7.6 μg/m^3^/AR 1kg AI/ha in the first 8 h, with a variation of 0.08 to 27 μg/m^3^/AR 1kg AI/ha. To produce the more pronounced rise around 48 h, air concentrations of LCT would have to average 41 μg/m^3^/AR 1kg AI/ha between 39 and 50 h after application. For the rise between 74 and 84 h after application, air concentrations of LCT would have to average 72 μg/m^3^/AR 1kg AI/ha. For the rise between 92 and 115 after application, the maximum air concentrations of LCT would have to reach 744 μg/m^3^/AR 1kg AI/ha.

With the 1000 sets of parametric values, 1000 time series of reconstructed absorbed dose values associated with good fits to the observed excretion time courses of CFMP were predicted using the model, for each applicator and each exposure scenario (exposure to LCT alone or combined with captan). The inadvertent hourly oral exposure scenario was retained, as it is the scenario giving the best results. Table 4 shows the reconstructed absorbed doses (*i*.*e*. median value with 95% confidence intervals (CI 95%) for the 1000 iterations) for the most exposed applicators (namely T101, T103, T106, T106b and T108) and for the exposure scenarios to LCT alone or combined with captan (E1 and E2 but also E3 for T101 and T106 and E5 for T106b). Table 5 shows the corresponding probabilities of exceeding the EFSA AOEL value of 630 ng/kg bw/d [45] in these most exposed applicators, as determined from the results of the Monte Carlo simulation process. For the applicators with the highest urinary concentrations, there was a probability of exceeding the AOEL at some points during the biological monitoring period. For example, there was a 72% probability that worker T103 exceeded the AOEL during the 0–24 h following LCT application.

**Table 4:** Absorbed doses reconstructed with the model and Monte Carlo simulations in the most exposed applicators.

Applicators and exposure scenario ^a^	Reconstructed absorbed dose in applicators (ng/kg bw/d) Median [95% confidence interval] of the 1000 reconstructions^b^					
	For a 0–24 h urine collection equivalent	For a 24–48 h urine collection equivalent	For a 48-72h urine collection equivalent	For a 72–96 h urine collection equivalent	For a 96–120 h urine collection equivalent	For a 120–144 h urine collection equivalent
T101 E1	136 [121–156]	354 [313–409]				
T101 E2 and E3	187 [165–218]	93 [85–100]	82 [74–89]	93 [85–103]	194 [178–216]	212 [191–230]
T103 E1	1347 [1205–1509]	1131 [1001–1290]	1135 [1011–1282]			
T103 E2	1178 [966–1383]	272 [226–322]				
T106 E1	1117 [995–1288]	242 [213–288]	746 [561–896]			
T106 E2 and E3	35 [30–38]	191 [171–223]	41 [37–48]	343 [295–392]	991 [838–1097]	196 [177–217]
T106b E1	245 [212–288]	113 [98–126]				
T106b E3	345 [288–397]	415 [355–477]	423 [380–504]			
T106b E2	452 [394–529]	409 [372–476]	----^c^			
T108 E1	874 [779–1005]	34 [28–43]	----^c^			
T108 E2	355 [313–405]	----^c^	62 [56–71]			

^a^ E1 = exposure to LCT alone following spraying in strawberry fields; E2 = exposure to LCT in combination with captan following spraying in strawberry fields; E3 = exposure to LCT in combination with captan following activities in treated fields.

^b^ As predicted by the toxicokinetic model and Monte Carlo simulations from temporal profiles of urinary CFMP. For each applicator and each exposure scenario E1, E2, E3, reconstructions of daily absorbed doses uses Monte Carlo simulations generating 1000 sets of parametric values and giving a fit to the observed data with a margin of error less than 5%. 95% confidence interval was obtained using bootstrap function.

^c^ Absorbed doses corresponding to a non-quantifiable value.

**Table 5:** Probabilities of exceeding the AOEL in the most exposed applicators.

Applicators and exposure scenario ^a^	Probability of exceeding the AOEL (%) ^b^ Based on the 1000 reconstructions					
	For a 0–24 h urine collection equivalent	For a 24–48 h urine collection equivalent	For a 48-72h urine collection equivalent	For a 72–96 h urine collection equivalent	For a 96–120 h urine collection equivalent	For a 120–144 h urine collection equivalent
T101 E1	13	35				
T101 E2 and E3	15	7	6	7	16	19
T103 E1	72	67	67			
T103 E2	65	29				
T106 E1	68	22	54			
T106 E2 and E3	2	21	3	31	63	28
T106b E1	68	22	54			
T106b E3	35	39	40			
T106b E2	39	35	12			
T108 E1	61	6	4			
T108 E2	33	0	18			

^a^ E1 = Exposure to LCT alone following spraying activities in strawberry fields; E2 = exposure to LCT in combination with captan following spraying activities in strawberry fields; E3 = exposure to LCT in combination with captan following activities in treated fields.

^b^ As predicted by toxicokinetic model and Monte Carlo simulations from temporal profiles of urinary CFMPs.

### Simulation of excretion in field workers performing weeding and picking, estimates of daily absorbed dose and probabilities of exceeding the AOEL

The toxicokinetic model was also used to reproduce the (three to four) consecutive 24-h urinary excretions in field workers other than applicators assigned to tasks (mainly weeding or strawberry picking) and estimate the corresponding daily absorbed doses. Table 6 shows the results of Monte Carlo simulations of median ☯95% CI] daily absorbed doses for all the field workers as reconstructed with the model from CFMP levels in 24-h urine collections in workers; these results consider 1000 sets of randomly generated parametric values giving a good fit to the observed data with a margin of error of less than 5%. Considering these absorbed doses, the corresponding probabilities of exceeding the AOEL for all field workers (performing weeding and strawberry picking) were determined as reported in Table 7. Results show the number of field workers with a probability of exceeding the AOEL (and the corresponding percentage) in the days following tasks in a treated field; it indicates that the probability of exceeding the AOEL value for non-applicator workers is very low. For example, only one worker (1% of workers) performing tasks in a field treated with LCT alone or in combination with captan had a 50–60% probability of exceeding the AOEL in the 24 h after starting a weeding or picking task; five field workers (6% of workers) had a 50–70% probability of exceeding the AOEL in the 48–72 h after starting one of these tasks.

**Table 6:** Absorbed doses reconstructed for field workers performing weeding and strawberry picking (all non-applicators).

.	Absorbed doses of LCT post-exposure in field workers ^a^ (ng/kg bw/day)			
	0–24 h	24–48 h	48–72 h	72–96 h
N of workers	82	82	82	34
Median [95% CI]	22 [21 – 24]	17 [16 – 18]	26 [25 – 27]	132 [129–136]
5^th^ Percentile	----^b^	----^b^	----^b^	----^b^
25^th^ Percentile	----^b^	----^b^	----^b^	----^b^
50^th^ Percentile	22 [21 – 24]	17 [16 – 18]	26 [25 – 27]	132 [129–136]
75^e^ Percentile	151 [148–154]	127 [124–129]	180 [176–184]	355 [349–363]
90^e^ Percentile	465 [456–474]	408 [398–418]	599 [587–611]	806 [787–824]
95^e^ Percentile	881 [861–903]	823 [800–846]	1080 [1059–1101]	1244 [1213–1272]

^a^ As predicted by the toxicokinetic model and Monte Carlo simulations from the daily urinary excretions of CFMP during the three days following tasks in fields treated with LCT.

^b^ Absorbed doses corresponding to a non-quantifiable value.

**Table 7:** Probabilities of exceeding the AOEL for field workers performing weeding and strawberry picking (all non-applicators).

Probability of exceeding the AOEL^a^	Number of workers N (%)			
	0–24 h urine collection	24–48 h urine collection	48–72 h urine collection	72–96 h urine collection
0%	36 (44)	32 (39)	30 (37)	10 (29)
>0–10%	26 (32)	32 (39)	33 (40)	5 (15)
>10–20%	8 (10)	7 (9)	3 (4)	11 (32)
>20–30%	7 (9)	5 (6)	5 (6)	4 (12)
>30–40%	3 (4)	4 (5)	3 (4)	1 (3)
>40–50%	1 (1)	2 (2)	3 (4)	3 (9)
>50–60%	1 (1)	0 (0)	3 (4)	0 (0)
>60–70%	0 (0)	0 (0)	2 (2)	0 (0)
>70–100%	0 (0)	0 (0)	0 (0)	0 (0)

^a^ As predicted by the toxicokinetic model and Monte Carlo simulations from the daily urinary excretions of CFMP during the three days following tasks in fields treated with LCT.

### Establishment of a biological reference value corresponding to the AOEL dose value

A biological reference value for CFMP was also derived using the toxicokinetic model by simulating a repeated exposure to the AOEL value of 0.00063 mg/kg bw/d until a steady state was reached (*i*.*e*., an hourly absorbed dose corresponding to 1/8 of the AOEL over an 8-h period per day repeated over five days) and determining the corresponding 24-h urinary CFMP levels. The median [95% CI] estimate of the 10 000 Monte Carlo simulations was 116 [113–119] ng/kg bw/d and 7.5 [7.3–7.7] μg/L, considering the calculated mean urine volume of 1.086 L in the study workers and the calculated mean body weight of 70.13 kg. Based on this biological reference value, 7% of the applicators and 1% of the workers performing weeding and strawberry picking had a probability of exceeding this biological reference value.

## Discussion

### Model development

The toxicokinetic model developed in this work using the data of Khemiri et al. [27, 28] on the time courses of CFMP and 3-PBA in the plasma and urine of volunteers orally and dermally exposed to LCT confirmed that the kinetics of these metabolites used as exposure biomarkers were similar to those of permethrin and cypermethrin metabolites [24, 25]. The oral absorption, distribution, metabolism and elimination rates of LCT modeled in the current work were found to be in the same order of magnitude as those of a toxicokinetic model developed to describe the kinetics of permethrin and cypermethrin metabolites [20, 50]. In particular, the conceptual and functional representation of the oral absorption of LCT in the toxicokinetic model was similar to that of the model used to simulate the kinetics of biomarkers of exposure to permethrin and cypermethrin metabolites in Côté et al. [20, 50]. However, to simulate the dermal absorption, this new model presented in the current work assumes that a portion of LCT dermally absorbed reaches the systemic circulation unchanged while another portion is in the metabolized form. This was conceptually and functionally modeled by introducing three skin sub-compartments to represent: i) LCT on the skin surface; ii) LCT within the skin structures; iii) the metabolites CFMP and 3-PBA formed within the skin structures. Although we did not identify any *in vitro* or *ex vivo* data specifically showing LCT metabolism in the skin, several authors have shown that pyrethroids are metabolized by carboxylesterases [51–53]. In addition, other authors have reported the presence of carboxylesterases in the internal structures of the skin [29, 54]. This presence of carboxylesterases in the skin may thus alter the kinetics of LCT when absorbed by the dermal route as compared to the oral route. It was not possible to obtain a good adjustment to (and prediction of) the available dermal kinetic data in volunteers of Khemiri et al. [28] without adding these skin sub-compartments and without considering metabolism in the skin.

Similar to the modeling of permethrin and cypermethrin kinetics, the modeling conducted in the present project confirmed that the oral absorption rate of LCT was faster than the elimination rate. For the dermal absorption of LCT, the toxicokinetic model indicates a very low absorption rate constant from the skin surface. The low dermal rate constant explains the low absorption fraction of LCT in the skin. A similar low absorption fraction through the skin was reported for other molecules such as malathion [55], parathion [56], chlorpyrifos [57], and carbaryl [21]. Nevertheless, our modeling of the kinetic data of Khemiri et al. [28] in volunteers dermally exposed to LCT under controlled conditions suggests a retention of the parent compound and metabolites in the skin.

Analysis of the urinary and blood profiles of metabolites in volunteers of the study of Khemiri et al. [27, 28] and modeling of these data show that LCT is absorbed and eliminated somewhat less rapidly following dermal exposure (T_max_ of 19 h) as compared oral exposure. This is reflected in the model parameters. A temporal follow-up of the amounts of metabolites recovered in serial urine collections of an individual (amounts per hour) would therefore allow to differentiate whether the time-to-peak excretion results from an oral or a dermal exposure. Nevertheless, the concentrations of CFMP and 3-PBA obtained in the blood and urine of volunteers assessed in the study of Khemiri et al. [28] following dermal exposure remained very low (C_max_ 0.004% of the dermal dose) and were close to the limit of detection, which may create uncertainty in the results. These results thus show that the urinary levels of LCT metabolites are very low following dermal exposure as compared to those observed after ingestion. This has a major impact on the interpretation of biomonitoring data in workers exposed to LCT and on inference of main exposure route.

### Predicted main exposure route in workers based on simulations of observed profiles

By considering a dermal absorption in the model, it was not possible to simulate adequately the observed data of Bossou et al. [39] on the time course of metabolites in the strawberry field operators applying LCT. The absorbed doses by the dermal route would have to be orders of magnitude higher than the AOEL to obtain concentrations values close to those observed in workers. The model skin penetration (obtained by adjustments to kinetic data in volunteers dermally exposed under controlled conditions) was too slow and too low to reproduce the observed urinary excretion time courses and time-to-peak levels in the applicators.

The model simulations of the observed time courses of LCT metabolites in the urine of applicators conducted in the current work rather confirmed previous modeling of excretion time courses of metabolites of other pyrethroids–permethrin and cypermethrin–in workers; this previous work suggested that workers were primarily exposed by inadvertent oral absorption (hand-to-mouth directly or indirectly) rather than by direct dermal absorption [31]. More specifically, simulation of an oral absorption scenario gave the best fits to the observed temporal profiles of exposure biomarkers in applicators, unlike a dermal absorption scenario where it was impossible to obtain convergence between the simulation points and the observed data; the skin absorption rate was too slow. Furthermore, simply by comparing observed daily amounts, an unrealistic dermal exposure dose (up to 500 times the AOEL) would be required to reach the urinary CFMP levels observed in the applicators and modeled in this study.

The modeling exercise we carried out to determine the dermal exposure dose that would be required to reach the observed peak in the temporal CFMP excretion profile for worker T106 would also necessitate touching the entire surface of 60 000 strawberry leaves (with a transfer rate of 2% according to Brouwer et al. [47]). Assuming that the surface is touched at 50% on average, the number of strawberry leaves handled by the worker would then amount to 120 000. This scenario is highly unlikely to occur during a shift, as it would be equivalent to touching 4 strawberry leaves per second for an 8 h shift. This scenario also ignores the wearing of protective gloves and the environmental degradation of LCT. The calculations done in the current work further imply dose accumulation over an entire shift, whereas volunteers exposed to Matador^®^ in the study of Khemiri et al. [28] received the full dose at once and left on the skin for 6 h, making the dermal absorption scenario unlikely in the assessed workers. Assuming CFMP urinary excretion rates 100 times lower (0.17 pmol/kg bw/h), the worker would still have to handle 1200 strawberry leaves. However, less than 1% of the measured excretion rate values of the followed operator workers were below 0.17 pmol/kg bw/h. These findings suggest that, even considering significantly reduced urinary excretion rates, absorption through the skin when handling strawberry leaves does not represent the main route-of-entry in our studied workers and remains implausible compared with the observed excretion data. Conversely, considering an hourly oral absorption scenario in the predictions for worker T106 would lead to a reconstructed median absorbed dose of 4.6 nmol (Fig 5C; predicted from the 1000 reconstructed profiles). Based on previous assumptions of the amount of LCT per strawberry leaf, the worker would only need to touch around 17 strawberry leaves to accumulate such a dose on his hands, always considering a no-glove scenario. Inadvertent hand-to-mouth exposure (or touching food with contaminated hands before eating) and thus oral absorption thus seems realistic. Such inadvertent hand-to-mouth exposure (or touching food with contaminated hands before eating) and thus oral absorption seems realistic even when workers wear protective gloves. In fact, contamination can occur simply by touching their gloves when removing them, or any other contaminated surface such as their clothing.

We also determined potential skin surface covered by approximately 124 μL of pure Matador^®^, or 300 mL of diluted Matador^®^ over a 6-h period. This type of exposure is possible for an applicator who prepares the solution before spreading the product in the field. Such an amount of pure Matador^®^ may seem minimal, but it is sufficient to cover the skin surface of both hands. It would thus probably not go unnoticed and remains unlikely given the protective equipment worn when preparing the solution.

Simulation of an inhalation exposure was also considered as an *a priori* plausible scenario in workers. However, reconstructed absorbed doses with the model considering an inhalation exposure scenario alone would necessitate very high airborne concentrations. In particular, Hatzilazarou et al. [58] undertook a study of atmospheric concentrations of endosulfan, dicofol, tetradifon, permethrin, bifenthrin, cypermethrin and deltamethrin, analyzed at specific time intervals (2, 6, 12 and 24 h, and 3, 6 and 12 days) post-application within greenhouses dedicated to geranium cultivation. Maximum concentrations were recorded 2 h after application but pyrethroid concentrations remained low to undetectable within 2 h of application. Permethrin, for example, had a concentration of 0.08 μg/m^3^/AR 1kg AI/ha. When comparing these results with simulations for LCT, it becomes clear that inhalation is not the predominant route of exposure for workers in the case of LCT.

### Dose reconstruction in strawberry field pesticide applicators and workers

The toxicokinetic model was used to reproduce the observed temporal profiles of CFMP in the urine of workers monitored in Bossou et al. [39] and establish the corresponding absorbed doses of LCT. Since 3-PBA is common to several pyrethroids, reconstruction of daily absorbed doses of LCT with the model was conducted using the more specific CFMP metabolite. Our modeling results showed that most field workers had estimated absorbed dose values that were below the AOEL of 630 ng/kg bw/d (0.63 μg/kg bw/d) established by EFSA [45]. In contrast, based on the model-reconstructed daily dose estimates during the biological monitoring period, some applicators had a probability of exceeding this AOEL value.

Similar to our work on the development and use of a pyrethroid toxicokinetic model to reconstruct absorbed doses in workers [20, 31, 59], Quindroit et al. [15] used a physiologically-based pharmacokinetic (PBPK) model to estimate pyrethroid exposure doses in the French National Nutrition and Health Survey (ENNS) cohort from urinary concentrations of five pyrethroid metabolites commonly measured in biomonitoring studies. The estimation was based on exposure to mixtures of four pyrethroids (deltamethrin, permethrin, cypermethrin and cyfluthrin). Their modeling approach takes into account cumulative exposure to pyrethroids, as some metabolites may be shared by several parent compounds, and interindividual variability in human metabolism. The global model of Quindroit et al. [15] is therefore a combination of seven PBPK models for the parent compounds (the *cis*- and *trans*-isomers of permethrin, cypermethrin, and cyfluthrin as well as deltamethrin) and five one-compartment models for metabolites. Global model parameters were determined from *in vitro* and *in vivo* animal and human data, as well as *in silico* predictions (*e*.*g*., for the tissue:blood partition coefficients). In our current study in workers, the reconstructed daily dose value was as high as 1347 ng/kg bw/d (1.35 μg/kg bw/d); in the previous study in vegetable crop workers exposed to cypermethrin, estimated daily dose levels were as high as 2400 ng/kg bw/d [20, 31]. Conversely, in the French ENNS cohort, the median daily dose was estimated to be 8.1 ng/kg bw/day for permethrin, 17.7 ng/kg bw/day for cypermethrin, 20.4 ng/kg bw/day for cyfluthrin, and 34.3 ng/kg bw/day for deltamethrin. This suggests that some workers in our study have peak exposures that greatly exceed the values for the general population. The modeling approach used by Quindroit et al. [15] is similar to that reported in Darney et al. [12] where a PBPK model for permethrin has been developed to reconstruct absorbed doses in the ENNS cohort. In particular, this model was based on our kinetic data on permethrin metabolites in volunteers exposed orally in controlled conditions [25]. In the study of Darney et al. [12], daily dietary intakes were estimated to be 400 ng/kg bw/day (intermediate estimate) with a lower estimate of 8.6 ng/kg bw/day and an upper estimate of 830 ng/kg/day. These values are higher than those predicted by the Quindroit et al. [15, 16] model.

### Determination of biological reference values

The toxicokinetic model developed in the current work was used to establish a biological reference value of 116 ng CFMP/kg bw/d in 24 h urine collections or 7.5 μg CFMP/L urine corresponding to the AOEL exposure limit value established by EFSA of 0.00063 mg/kg bw/d. This biological reference value takes into account variability in the kinetic parameters of LCT and its biomarkers of exposure. The CFMP excretion levels observed in the workers monitored by Bossou et al. [39] were thus compared to this biological reference value calculated in the present work. While several workers showed biological levels that exceeded the values observed in the general population, 7% of the applicators had a probability of exceeding this biological reference value and 1% of the other workers assigned to weeding and picking.

Recently, in the framework of the European Human Biomonitoring for Europe Initiative (HBM4EU) Consortium, a methodology for setting human biomonitoring guidance values for the general population (HBM-GVGenPop) has been proposed [19, 40, 60] and implemented for various priority substances, including several pyrethroids [43]. Similar to Health Canada’s biomonitoring equivalents (BEs), the HBM-GVGenPop are biological values, mainly urinary levels of contaminants or their metabolites, established from toxicological and epidemiological data that can be used for comparison with biomonitoring data in the general population. Such a value was derived for the metabolite CFMP (otherwise known as ClF_3_ CA) of LCT [19, 40–44]. This HBM-GVGenPop represents a urinary level that serves as an estimate of internal exposure and corresponds to a toxicological guidance or reference value, *i*.*e*., the acceptable daily intake (ADI) in the case of dietary exposure to pesticides. A HBM-GVGenPop of 14 μg/L in adults was established for CFMP (ClF_3_CA), based on the ADI of 0.0025 mg/kg bw and a 21% recovery of the metabolite CFMP (ClF_3_CA) in urine in our previous study in volunteers (Khemiri et al. [27]). This biological reference value of 14 μg/L for CFMP is in the same order of magnitude as that derived by our modeling approach (7.5 μg/L). The highest 95^th^ percentile values of CFMP (ClF_3_ CA) reported for the populations covered by the HBM4EU harmonized studies were 1.05 μg/L CFMP (ClF_3_CA) for adults in a study in Israel and 1.28 μg/L for children in a study in the Netherlands, which are lower than the HMB-GVGenPop for ClF_3_CA established by the HBM4EU Consortium [43, 61]. In the present study, the geometric mean urinary CFMP concentration values calculated in μg/L for all workers were 1.17 μg/L, and the median and 95^th^ percentile were 0.97 and 12.5 μg/L. While the 95^th^ percentile of CFMP concentrations for workers assessed in Bossou et al. [39] and modeled in the current study did not exceed the HBM-CVGenPop of 14 μg/L, some workers had 3-PBA levels that exceeded the "screening value" of 4.8 μg/L established by the European Consortium. For applicators of our study specifically, the median CFMP concentration was 1.36 μg/L and the 95^th^ percentile was 19.1 μg/L; 7% of applicators had a CFMP concentration value that exceeded the HBM-CVGenPop of 14 μg/L.

### Limits

The determination of model parameters was carried out on a small number of participants, which may limit the representation of inter-individual variability. Nevertheless, to counter this limitation, the reconstruction of absorbed doses was based on a lognormal distribution of the parameters determined in the volunteers. With these conditions, the sets of parametric values obtained by Monte Carlo simulations and the lognormal distributions make it possible to virtually represent a larger population than just the volunteers under study. With such conditions, the model was able to reconstruct absorbed doses in farmworkers exposed to LCT.

## Conclusions

Overall, the LCT model allowed determining the relationship between absorbed doses of this specific pyrethroid and its urinary metabolite levels. Despite its relative simplicity, the strength of this modelling method lies in the fact that the data come from a human study carried out in a controlled environment, thus reducing the approximations generated by the use of animal or *in vitro* studies to determine model parameters. The model was able to reconstruct absorbed doses in farmworkers exposed to LCT on the basis of the more specific CFMP metabolite. The 3-PBA metabolite was not used, as it is not specific to LCT, which could have led to misrepresentations of actual exposure. Furthermore, dose reconstruction enabled us to deduce the main route of exposure in the studied workers. Simulation of an oral exposure scenario gave the best fits to the observed temporal urinary profiles and plausible reconstructed absorbed doses in agricultural workers. Modelling of the urinary kinetic profiles of CFMP in applicators led to the conclusion that the dermal and inhalation routes of exposure were negligible sources of absorption compared with the oral route. Skin absorption simulations were unable to represent the observed temporal urinary profiles of CFMP in applicators, as absorption processes are too slow and low. Inhalation simulations were able to represent temporal profiles but led to LCT concentrations in air that were far too high to represent reality. Therefore, the modelling led to the conclusion that, although cutaneous and inhalation exposure routes may be present, they are not the main exposure routes contributing to absorbed levels in the assessed workers. In addition, the reconstruction of absorbed doses in workers has made it possible to establish probabilities of exceeding the AOEL reference value. Unlike approaches attempting to predict an exact absorption value for a worker, a probabilistic approach to exceeding a reference value was used and deemed more appropriate, given that it is not possible to know a person’s individual parameters. Thus, an approach based on several sets of parametric values that adequately represent the kinetic urinary profiles of CFMP and the associated absorbed doses served to determine the probability of exceeding a reference value in exposed workers. Finally, in this study, only one biomarker of exposure was monitored (CFMP). With a view of developing and advancing biomonitoring, it would be interesting to apply these methods in the context of multiple exposure to various contaminants. It would also be relevant to assess the relative contribution of hand-to-mouth oral exposure versus dietary exposure to pesticide residues.

## Supporting information

S1 Appendix Summary of studies used in the development of the LCT model and absorbed dose reconstruction. (DOCX)

S2 Appendix Differential equations representing the toxicokinetic model of LCT and its metabolites. (PDF)

S1 Fig Algorithm used for the determination of the model parametric values. (TIF)

S2 Fig Algorithm used for dose reconstruction in workers. (TIF)
