# Supplementary material for: Toxicokinetic model of the pyrethroid pesticide lambda-cyhalothrin, main exposure route and dose reconstruction predictions in agricultural workers
Source: PLoS One. 2024 Oct 23;19(10):e0309803. doi: 10.1371/journal.pone.0309803 (PMC11498739; doi:10.1371/journal.pone.0309803)
Supplement: S1 Appendix — (DOCX) [file pone.0309803.s001.docx]

**S1 Appendix**

**Summary of studies used in the development of the LCT model and absorbed dose reconstruction**

**Published data used for LCT model development**

The published data of Khemiri et al. [1, 2] – from our laboratory – on the blood and urinary profiles of the metabolites CFMP and 3-PBA observed in volunteers exposed orally and dermally to LCT were used to build the toxicokinetic model and determine its parameters. In brief, in the latter study, four men and three women were recruited on a voluntary basis. Recruitment occurred between 22-09-2015 and 16-11-2015 (d/m/y). The volunteers were between 20 and 41 years of age, weighed between 53 and 93 kg (average of 70 kg), and were between 154 and 186 cm tall. They were nonsmokers, in good health, and not taking any medication. During the three days before dosing, participants were not allowed to eat fruits, vegetables or nuts from the market, as contaminated food is generally known to be the primary route of pesticide intake in the general population [3]. They were given organic food as a substitute. During the biomonitoring period, meals and snacks prepared with certified organic ingredients were also provided to each participant. They were also asked to avoid tea, herbal tea, alcohol, and medications during this period; the latter two can affect the metabolism of certain chemicals by interfering with enzyme activity [4, 5]. In addition, a questionnaire was administered to ensure that participants had not been exposed to pyrethroids in the month prior to the first experimental dosing, except possibly through the diet.

For the oral exposure, the seven volunteers received a dose of LCT of 0.025 mg/kg bw dissolved in 2 mL of organic olive oil. They were then asked to drink 100 mL of water. The mouthpiece used for dosing was rinsed with pure oil and the oil was administered to the participants, along with another 100 mL of water. For the dermal exposure, six of the seven volunteers were subsequently exposed about one month later to a Matador^®^ formulation at a dose corresponding to 0.25 mg/kg bw LCT. The product was applied to 40 cm^2^ of skin on one forearm and left for 6 h. A 20 x 2 cm cardboard frame was placed on the forearm of each volunteer to delineate the application area. The dose was applied with a pipette and a polypropylene tip. The treated area was left uncovered and washed 6 h after application with soap and water. This type of application is therefore similar to that of exposed workers. In order to establish the kinetics of metabolites in blood, serial blood samples were taken by venipuncture from the arm before exposure (t = 0, *i.e.* 30 minutes before pesticide administration) and at fixed times over a 72-h period after exposure, *i.e.* 0.5, 1, 1.5, 2, 4, 6, 8, 10, 24, 48, and 72 h after administration (n = 12 samples per individual, per exposure) To determine the urinary excretion kinetics of the metabolites, complete urine voids were also collected in separate, clearly labeled polypropylene Nalgene^®^ bottles at fixed times, namely at times -3 - 0 h pre-exposure and 0 - 3, 3 - 6, 6 - 9, 9 - 12, 12 - 24, 24 - 36, 36 - 48, 48 - 60, 60 - 72, 72 - 84 h post-administration (n = 11 collections per individual, per exposure).

**Published biomonitoring data in agricultural workers used for dose reconstruction**

The biomonitoring data from the field study of Bossou et al. [6] on the kinetics of CFMP served to reconstruct absorbed doses of LCT in strawberry farmworkers using the toxicokinetic model developed in the current work. In brief in the latter study, a total of 87 workers assigned to different tasks – application of pesticides, weeding, strawberry picking – were recruited, and evaluated under their usual working conditions. Recruitment occurred between 14-05-2019 and 12-09-2020 (d/m/y). The target workers were exposed to LCT alone, or alternatively, in combination with captan. However, as shown in the study of Bossou et al. [6], co-exposure to captan had no significant effect on the urinary metabolite profiles of workers exposed to LCT. Recruited workers performing tasks in the strawberry fields were asked to provide a first full 24-h urine collection prior to an exposure episode to establish baseline exposure levels as well as two consecutive complete 24-h urine collections following an episode of LCT spaying alone or in combination with captan or tasks in treated fields (weeding and strawberry picking). Recruited applicators were also asked to collect urine over a 24-h period prior to the onset of LCT spraying alone or mixed with captan, in order to establish baseline exposure; urine over this 24-h preexposure period was collected in a single bottle. However, for applicators specifically, in a second step, they were asked to collect all urine voids (micturition) during three days after spraying, using separate bottles. Each applicator provided all urine voided over a three-day period after application of a pesticide formulation containing LCT alone (E1) and after application of formulations containing LCT mixed with captan (E2). Some applicators also performed tasks in the area treated with LCT and captan in the days following spraying and thus also provided all urine voided over three days after entering a treated field (E3). Participants were asked not to change their work habits during the biomonitoring period.

Potential determinants of metabolite levels used as biomarkers of exposure or potential confounders were assessed by questionnaire in addition to co-exposure to LCT and captan. These included the main work tasks performed, work practices and hygiene, personal protective equipment as well as personal factors and lifestyle habits [7]. Main questions were the same as those used in a previous work on cypermethrin exposure in workers [8]. Members of the team also conducted field observations during the first day of exposure.

**S1 References**

1. Khemiri R, Côté J, Fetoui H, Bouchard M. Documenting the kinetic time course of lambda-cyhalothrin metabolites in orally exposed volunteers for the interpretation of biomonitoring data. Toxicol Lett. 2017 Jul 5;276:115-121. doi: 10.1016/j.toxlet.2017.05.022. Epub 2017 May 21. PMID: 28539253.
2. Khemiri R, Côté J, Fetoui H, Bouchard M. Kinetic time courses of lambda-cyhalothrin metabolites after dermal application of Matador EC 120 in volunteers. Toxicol Lett. 2018 Oct 15;296:132-138. doi: 10.1016/j.toxlet.2018.08.008. Epub 2018 Aug 16. PMID: 30120931.
3. Schettgen T, Heudorf U, Drexler H, Angerer J. Pyrethroid exposure of the general population-is this due to diet. Toxicol Lett. 2002 Aug 5;134(1-3):141-5. doi: 10.1016/s0378-4274(02)00183-2. PMID: 12191872.
4. Choi J, R L Rose, Ernest H. In vitro human metabolism of permethrin: the role of human alcohol and aldehyde dehydrogenases. *Pesticide Biochemistry and Physiology. 2002* Nov;74(3): 117-128. doi : 10.1016/S0048-3575(02)00154-2
5. Guéguen Y, Mouzat K, Ferrari L, Tissandie E, Lobaccaro JM, Batt AM, et al. Les cytochromes P450: métabolisme des xénobiotiques, régulation et rôle en clinique [Cytochromes P450: xenobiotic metabolism, regulation and clinical importance]. Ann Biol Clin (Paris). 2006 Nov-Dec;64(6):535-48. French. PMID: 17162257.
6. Bossou YM, Côté J, Mahrouche L, Mantha M, El Majidi N, Furtos A, et al. Excretion time courses of lambda-cyhalothrin metabolites in the urine of strawberry farmworkers and effect of coexposure with captan. Arch Toxicol. 2022 Sep;96(9):2465-2486. doi: 10.1007/s00204-022-03310-5. Epub 2022 May 14. PMID: 35567602.
7. Bossou YM, Côté J, Morin É, Dumais É, Bianchi C, Bouchard M. Assessing the impact of coexposure on the measurement of biomarkers of exposure to the pyrethroid lambda-cyhalothrin in agricultural workers. Int J Hyg Environ Health. 2023 Jun;251:114194. doi: 10.1016/j.ijheh.2023.114194. Epub 2023 Jun 6. PMID: 37290330.
8. Ratelle M, Côté J, Bouchard M. Time courses and variability of pyrethroid biomarkers of exposure in a group of agricultural workers in Quebec, Canada. Int Arch Occup Environ Health. 2016 Jul;89(5):767-83. doi: 10.1007/s00420-016-1114-x. Epub 2016 Feb 1. PMID: 26831869.
