# Supplementary material for: Toxicokinetic model of the pyrethroid pesticide lambda-cyhalothrin, main exposure route and dose reconstruction predictions in agricultural workers
Source: PLoS One. 2024 Oct 23;19(10):e0309803. doi: 10.1371/journal.pone.0309803 (PMC11498739; doi:10.1371/journal.pone.0309803)
Supplement: S2 Appendix — (PDF) [file pone.0309803.s002.pdf]

S2 Appendix

Differential equations representing the toxicokinetic model of lambda-cyhalothrin and its metabolites

The differential equation system can be represented as: $\vec{X}'(t) = A\vec{X}(t)$

where X' are the time-drives of X and A is the matrix of constant coefficients (the transfer coefficients between the boxes).

The solution of X'=AX can be written as: $\vec{X}(t) = a1e^{\lambda 1t}\vec{V}1 + a2e^{\lambda 2t}\vec{V}2 + \cdots + ane^{\lambda nt}\vec{V}n$

where V_n are the eigenvectors and λ_n are the eigenvalues of the matrix A. a_n are constants.

The values of a_n are found by setting the initial conditions. For t=0, we have that:

$\vec{X}(0) = a1\vec{V}1 + a2\vec{V}2 + \cdots + an\vec{V}n.$

This system can also be solved by a matrix operation. Indeed, by putting the system in matrix form, we obtain: $\vec{X}(0) = \vec{a}\vec{V}$.

The solution to this system is: $\vec{V}^{-1}\vec{X}(0) = \vec{a}$.

Specifically, the LCT model system is:

$$\vec{X}'(t) = \begin{bmatrix} D(t)/dt \\ GI(t)/dt \\ RT(t)/dt \\ M_{non_{monitored}}(t)/dt \\ B(t)/dt \\ S(t)/dt \\ D_{in}(t)/dt \\ MD(t)/dt \\ M(t)/dt \\ U(t)/dt \\ F(t)/dt \end{bmatrix}$$

$$\vec{X}(t) = \begin{bmatrix} D(t) \\ GI(t) \\ RT(t) \\ M_{non_{monitored}}(t) \\ B(t) \\ S(t) \\ D_{in}(t) \\ MD(t) \\ M(t) \\ U(t) \\ F(t) \end{bmatrix}$$

$$A = \begin{bmatrix}
-k_{DD_{in}} & 0 & 0 & 0 & 0 & 0 & 0 & 0 & 0 & 0 & 0 \\
-k_{abs_oral} & 0 & 0 & 0 & 0 & 0 & 0 & 0 & 0 & 0 & 0 \\
-k_{abs_inh} & 0 & 0 & 0 & 0 & 0 & 0 & 0 & 0 & 0 & 0 \\
0 & 0 & 0 & 0 & k_{BM_NO} & 0 & k_{DinM_NO} & 0 & 0 & 0 & 0 \\
0 & k_{abs_oral} & k_{abs_inh} & 0 & -(k_{BS} + k_{BM} + k_{BM_NO}) & k_{SB} & k_{DinB} & 0 & 0 & 0 & 0 \\
0 & 0 & 0 & 0 & k_{BS} & -k_{SB} & 0 & 0 & 0 & 0 & 0 \\
k_{DD_{in}} & 0 & 0 & 0 & 0 & 0 & -(k_{DinB} + k_{DinMD} + k_{DinM_NO}) & 0 & 0 & 0 & 0 \\
0 & 0 & 0 & 0 & 0 & 0 & k_{DinMD} & -k_{MDM} & 0 & 0 & 0 \\
0 & 0 & 0 & 0 & k_{BM} & 0 & 0 & k_{MDM} & -(k_{MU} + k_{MF}) & 0 & 0 \\
0 & 0 & 0 & 0 & 0 & 0 & 0 & 0 & k_{MU} & 0 & 0 \\
0 & 0 & 0 & 0 & 0 & 0 & 0 & 0 & k_{MF} & 0 & 0
\end{bmatrix}$$
