# Supplementary figures and images for: Toxicokinetic model of the pyrethroid pesticide lambda-cyhalothrin, main exposure route and dose reconstruction predictions in agricultural workers
Source: PLoS One. 2024 Oct 23;19(10):e0309803. doi: 10.1371/journal.pone.0309803 (PMC11498739; doi:10.1371/journal.pone.0309803)

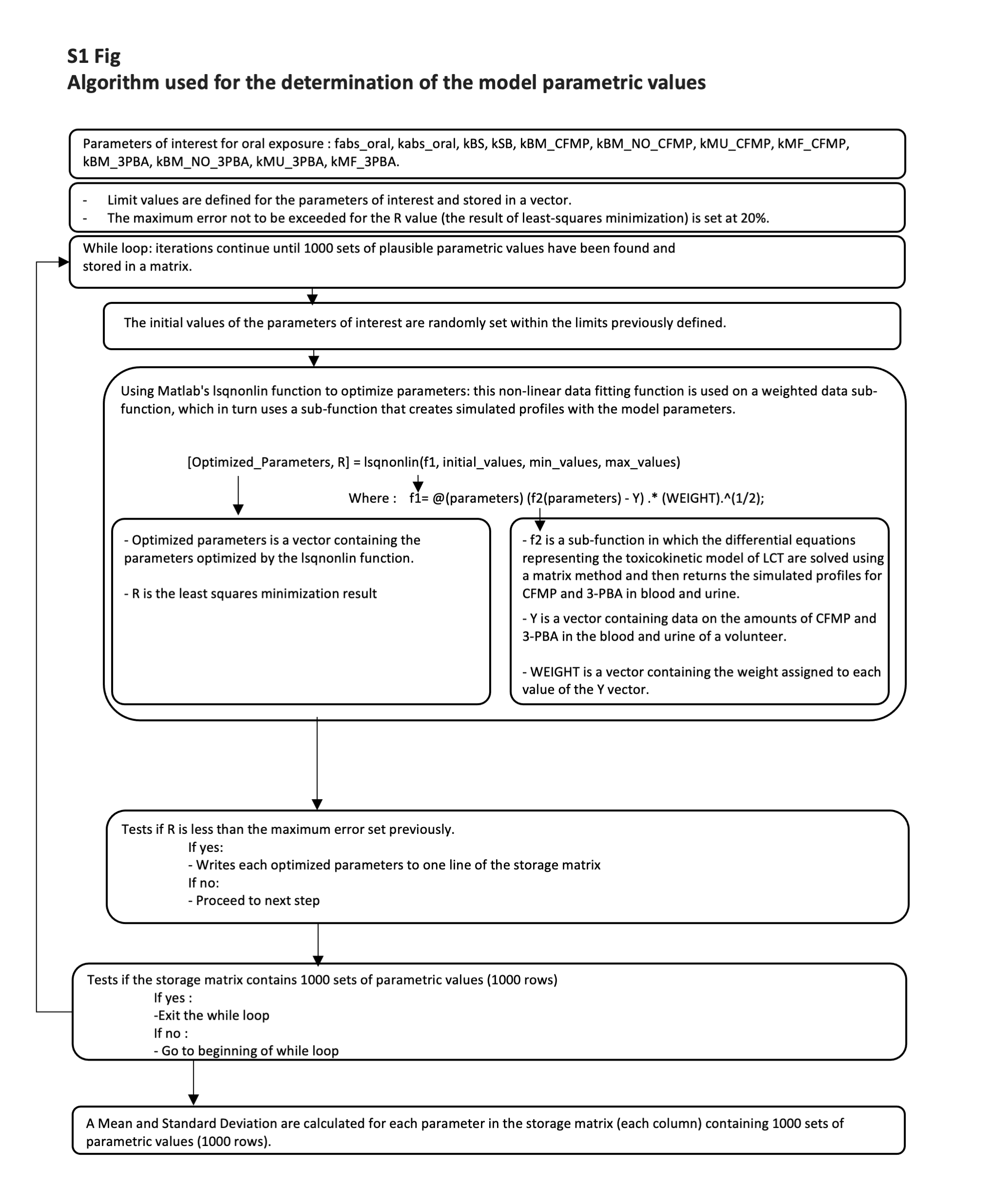

Supplement: S1 Fig — (TIF) [file pone.0309803.s003.tif]

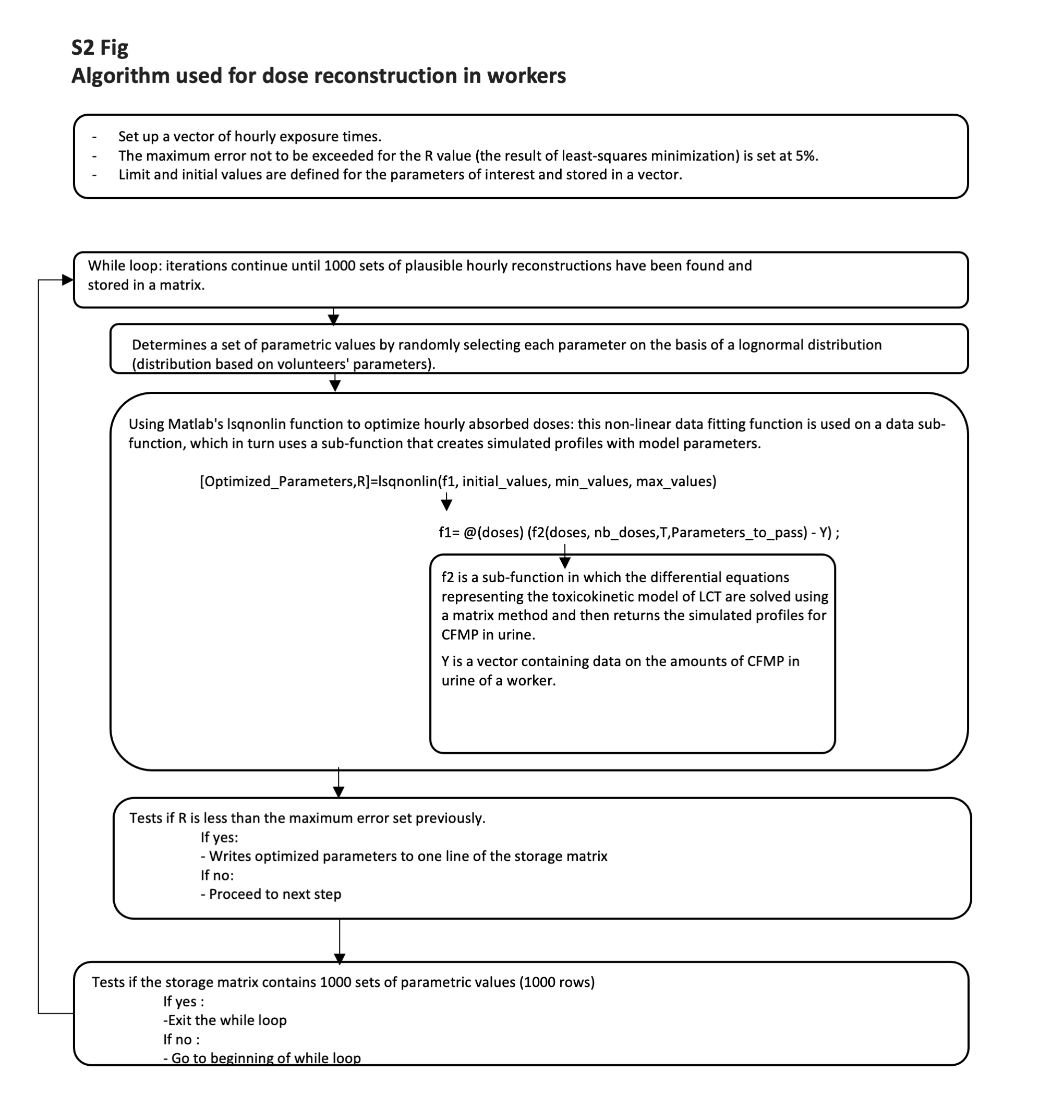

Supplement: S2 Fig — (TIF) [file pone.0309803.s004.tif]
